# A Patient with Rapidly Progressive Proptosis

**DOI:** 10.5334/jbr-btr.1325

**Published:** 2017-09-01

**Authors:** Sven Dekeyzer

**Affiliations:** 1 OLV-Ziekenhuis Aalst, BE

**Keywords:** proptosis, MRI, orbital pseudotumor, idiopathic orbital inflammatory syndrome, myositis

A 76-year-old woman was referred to the emergency department by her ophthalmologist because of left-sided ocular pain, redness, increased lacrimation and proptosis. The complaints had developed rapidly over several days’ time. Computed Tomography (CT) of the brain was performed which showed contrast-enhancing fusiform thickening of the left-sided medial and lateral rectus muscles, and to a lesser extent of the superior rectus muscle, as well as proptosis (Figures 1, 2). MRI confirmed the earlier seen CT changes and ruled out carotid-cavernous fistula or other cavernous sinus pathology (Figure 3). Thyroid function tests were normal. Based on the imaging findings a diagnosis of idiopathic orbital inflammatory syndrome was made and the patient was started on steroids, under which her symptoms improved rapidly.

**Figure 1:**
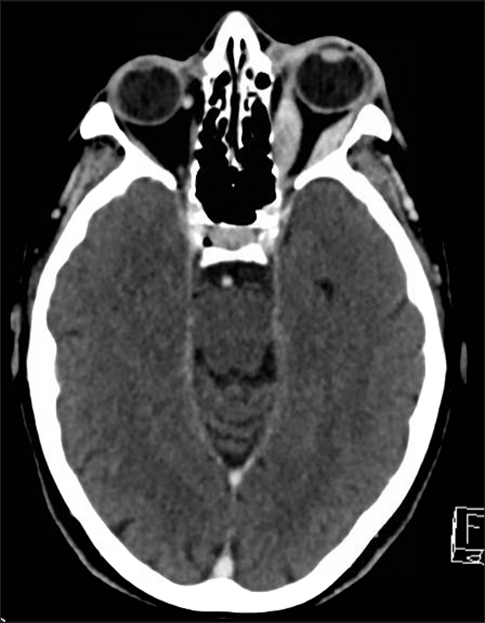
Contrast-enhanced axial CT of the brain shows fusiform and contrast-enhancing thickening of the left lateral and medial rectus muscles.

**Figure 2:**
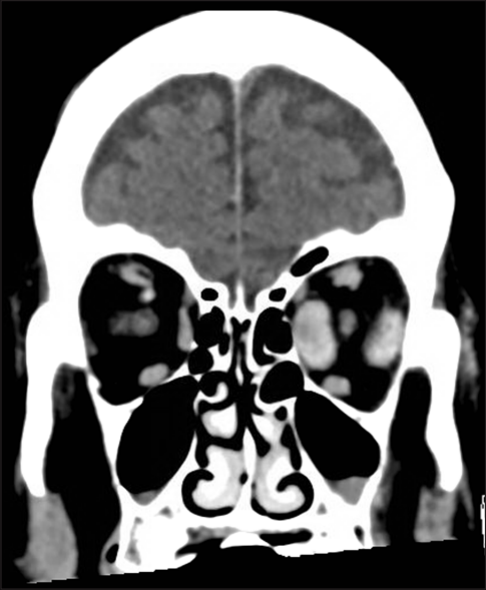
Coronal reconstructions of contrast-enhanced brain CT show fusiform thickening of the left medial and lateral rectus muscles as well as slight thickening of the left superior rectus muscle.

**Figure 3:**
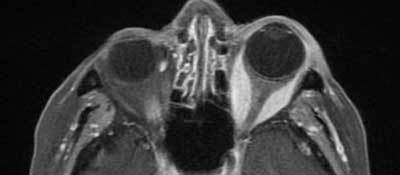
Contrast-enhanced fat suppressed axial T1-weighted MR-images of the orbit confirm fusiform thickening of the left medial and lateral muscles. No extension of intra-orbital changes into the cavernous sinus is seen.

## Discussion

Idiopathic orbital inflammatory syndrome (IOIS), also known as orbital pseudotumour, is a benign, non-neoplastic, non-infectious inflammatory disease of the orbit without identifiable local or systemic causes. It is the third most common orbital disease after Graves’ disease and lymphoproliferative disorders, and accounts for approximately 5–10% of orbital disorders [1]. IOIS can occur at any age, but most commonly in middle-age individuals. There is no race or sex predilection. Clinical presentation can be acute or subacute. Symptoms depend on the location and degree of inflammatory changes and include pain, redness, edema, proptosis, ptosis, reduced ocular motility, diplopia and/or chemosis. Severely impaired vision occurs in about 15% of cases. IOIS is typically unilateral and the presence of bilateral disease suggests an underlying systemic disease.

The diagnosis of IOIS is mainly one of exclusion and the most important diagnostic tool in IOIS is MR-imaging (MRI). The abnormalities seen on MRI vary depending on the involved site, which include the lacrimal gland (dacryoadenitis), the extraocular muscles (myositis), the preseptal or postseptal orbital fat (periorbital cellulitis and orbital cellulitis respectively), the optic nerve and nerve sheath (perineuritis) and the sclera, uvea and/or Tenon’s capsule ([epi-]scleritis/uveitis). Multiple sites can be simultaneously involved and the MR-abnormalities usually consist of swelling and contrast-enhancement of the structures involved. In our patient only the extra-ocular muscles were involved.

The etiology of IOIS remains unclear, but IOIS is presumed to be autoimmune in origin with viral, genetic and environmental factors proposed as possible triggers. The treatment of IOIS consists of corticosteroids given over a period of months. The response to therapy is generally rapid and favourable, but relapses are common. In refractory cases, radiotherapy may be used.
